# A whole-body physiologically based pharmacokinetic (WB-PBPK) model of ciprofloxacin: a step towards predicting bacterial killing at sites of infection

**DOI:** 10.1007/s10928-016-9486-9

**Published:** 2016-08-30

**Authors:** Muhammad W. Sadiq, Elisabet I. Nielsen, Dalia Khachman, Jean-Marie Conil, Bernard Georges, Georges Houin, Celine M. Laffont, Mats O. Karlsson, Lena E. Friberg

**Affiliations:** 1 Department of Pharmaceutical Biosciences, Uppsala University, Box 591, 75124 Uppsala, Sweden; 2 CVMD iMED, DMPK, Astrazeneca, Mölndal, Sweden; 3 INRA, Toxalim, Toulouse, France; 4 Universite de Toulouse, Toulouse, France; 5 Laboratoire de Pharmacocinetique et Toxicologie Clinique, Hospital Purpan, Institut Federatif de Biologie, Toulouse, France; 6 Pole d’Anesthesie-Reanimation, Hopital Rangueil, Toulouse, France

**Keywords:** Physiologically-based pharmacokinetic, Antibiotic, Modeling, Bacterial infection, Pharmacokinetic-pharmacodynamic, Fluoroquinolone, Informative priors, NONMEM

## Abstract

The purpose of this study was to develop a whole-body physiologically based pharmacokinetic (WB-PBPK) model for ciprofloxacin for ICU patients, based on only plasma concentration data. In a next step, tissue and organ concentration time profiles in patients were predicted using the developed model. The WB-PBPK model was built using a non-linear mixed effects approach based on data from 102 adult intensive care unit patients. Tissue to plasma distribution coefficients (Kp) were available from the literature and used as informative priors. The developed WB-PBPK model successfully characterized both the typical trends and variability of the available ciprofloxacin plasma concentration data. The WB-PBPK model was thereafter combined with a pharmacokinetic–pharmacodynamic (PKPD) model, developed based on in vitro time-kill data of ciprofloxacin and *Escherichia coli* to illustrate the potential of this type of approach to predict the time-course of bacterial killing at different sites of infection. The predicted unbound concentration–time profile in extracellular tissue was driving the bacterial killing in the PKPD model and the rate and extent of take-over of mutant bacteria in different tissues were explored. The bacterial killing was predicted to be most efficient in lung and kidney, which correspond well to ciprofloxacin’s indications pneumonia and urinary tract infections. Furthermore, a function based on available information on bacterial killing by the immune system in vivo was incorporated. This work demonstrates the development and application of a WB-PBPK–PD model to compare killing of bacteria with different antibiotic susceptibility, of value for drug development and the optimal use of antibiotics .

**Electronic supplementary material:**

The online version of this article (doi:10.1007/s10928-016-9486-9) contains supplementary material, which is available to authorized users.

## Introduction

The time-course of the distribution of an antibiotic into an infected tissue can be of critical importance for successful therapy of the infection. Based on the physicochemical properties of a drug and its interaction with different transporters, the rate and extent of distribution differs between tissues [1, 2]. Sub-therapeutic exposure of an antibiotic at the site of infection may not only result in treatment failure but also emergence of resistance, while higher than therapeutic levels may result in toxicity. Typically, when pharmacokinetic–pharmacodynamic (PKPD) relationships of antibiotics are evaluated, the bacterial killing is assumed to be dependent on a summary variable of the pharmacokinetic (PK) profile in plasma (e.g. peak plasma concentration, area under the plasma concentration–time curve or time period that the plasma concentration exceeds the MIC) [3]. Using the plasma concentration to monitor and drive the antimicrobial effect is practical, but the plasma concentration is not always reflecting the biophase antimicrobial concentration. In addition, the dynamics of the concentrations during the initial treatment period may be critical for a successful outcome. Overlooking that the time-course of concentration in the infected tissue is what is driving the bacterial killing could lead to suboptimal dosing in patients. In the era of increasing antibiotic resistance, new therapies need to be explored for previously untested types of infections. To efficiently get an idea of the potential of these therapies it is important to understand the distribution and exposure time profile of antimicrobials in the target tissues.

Whole body physiologically based pharmacokinetic (WB-PBPK) models encompass the distinct feature of describing the distribution of a drug in different tissues and blood in a mechanistic way [4–6]. Such models have long been used in toxicological risk assessment for different environmental contaminants [7]. In recent years they have been increasingly applied in different stages of drug discovery and development, and also recognized by regulatory authorities as a valuable tool [1, 7–12]. WB-PBPK models require two types of input data; physiological parameters such as blood flows, organ volumes, tissue compositions and drug related parameters like plasma protein binding, clearance and tissue to plasma partition coefficients (Kp). The Kp values reflect the distribution in the tissue in comparison to plasma at steady state. It is tedious, time and resource extensive to measure these values for each tissue experimentally, and based on the physicochemical properties of a compound there are different methods proposed to predict Kp values [10, 13–15]. Valuable information on Kp values may also be collected from in vivo experiments reported in the literature especially for antibiotics which are used prophylactically before soft tissue surgery due to different pathological indications.

During recent years, there have been different PKPD-models proposed to understand and quantify the time-course of bacterial killing [3, 16]. Such models may subsequently be linked to a PK model to predict the bacterial killing following different antibiotic dosing regimens [17, 18]. In this way, complex kinetic profiles, such as multiple compartment kinetics (e.g. gentamicin) and formation from prodrugs (e.g. colistin) can be mimicked without a range of extensive new time-kill experiments. Typically, the unbound concentration in the central compartment (e.g. plasma) has been used to drive the killing of the bacteria in the PKPD-models, i.e. a sepsis situation has been simulated.

In a patient the clinical outcome of the therapy will also be influenced by the presence of the immune system. Guo et al. has proposed a model for the quantitative impact of neutrophils on bacterial clearance in mice where the bacterial killing was dependent on the grade of immunosuppression, i.e. the absolute neutrophil count [19]. Further, Drusano et al. have shown in mice that the bacterial clearance by the immune cells is saturable and a model was proposed where the bacterial killing was dependent on the bacterial burden [20]. The time-course of the efficiency of the immune system to clear the infection is however most likely dependent on both the number of bacteria at the site of infection and the competence of the immune system.

Ciprofloxacin belongs to the fluoroquinolone class of antimicrobials and has been extensively used the last two decades in the clinic because of its broad spectrum against bacteria [21]. Fluoroquinolones are associated with rapid emergence of resistance and hence appropriate therapeutic use of this class is prime to keep their effectiveness. As ciprofloxacin has been used for many years it is also possible to collect information on Kp values based on clinical samples. Therefore, ciprofloxacin can serve as an example on how in vitro, in vivo and literature information can be integrated into a whole-body PBPK model to predict unbound concentrations in different tissues and organs. Recently a PKPD model was developed by Khan et al. based on data from time-kill experiments with constant ciprofloxacin experiments [22]. The model describes the growth and killing kinetics of a wild-type *Escherichia coli* strain, and six well-characterized mutants thereof, with different degree of resistance, during exposure to various ciprofloxacin concentrations.

In this study we aimed to develop a WB-PBPK model for predictions of the tissue/organ concentration–time profiles of ciprofloxacin in patients. In a next step the model was combined with a PKPD model, describing bacterial growth and ciprofloxacin killing kinetics, as well as a model characterizing the effect of the immune system. The combined model was used to illustrate the potential value of this approach to predict the time-course of bacterial killing for infections with *E. coli* strains with different levels of resistance.

## Methods

### WB-PBPK model building

A WB-PBPK model for ciprofloxacin was developed based on plasma concentration data from 102 adult patients admitted to the Intensive Care Unit (ICU) for different indications. The data has earlier been used to develop a 2-compartment population PK model [23]. There were 27 women and 75 males in the patient population with an average±SD total body weight of 77±16 kg and an average age of 60±17 years. Measured creatinine clearance (CRCL) was 82±51 ml min^−1^. All patients were mechanically ventilated and were on ciprofloxacin infusion therapy during their stay in the ICU. Among the 102 patients included in the study 86 received 400 mg ciprofloxacin as a 1 h infusion twice a day, 9 patients received 400 mg three times a day, 6 patients received 200 mg twice a day (30 min infusion), and 1 patient received 600 mg twice a day [23]. Duration of treatment ranged from 3 to 21 days (average = 12 days). In total 588 plasma concentrations were available, with on average 5.8 samples from each patient. Samples were taken on different days where one occasion was defined to be one dose administration interval (average = 3.1 occasions/patients).

A population modeling approach was applied to fit WB-PBPK models to the plasma PK data using NONMEM version 7.3 with first order conditional estimation [24]. All plasma concentrations from patients were transformed into natural logarithms before the data analysis. An additive error model was used on log-transformed data. During the estimation process the only dependent variable was the plasma concentrations.

Typical population ciprofloxacin tissue to plasma distribution coefficients (Kp) for 10 different tissues including lung, muscle, kidney and adipose were taken from clinical studies available in the literature and used as informative priors while estimating the tissue Kp values [25–32]. Kp values are determined from the ratio of total tissue and plasma concentrations and described according to Eq. (1).

1 $$\rm K_{\rm p} \, = \, \rm C_{\rm Tissue} /C_{\rm Plasma}$$

when a Kp value was not directly reported from a study (spleen, heart and brain) the value was calculated by using the ratio of the reported tissue and plasma concentrations. A “Rest” compartment represented other parts of the body for which no Kp is defined, and for this compartment the typical Kp was set to the average of all Kp values for other tissues.

**Fig. 1:**
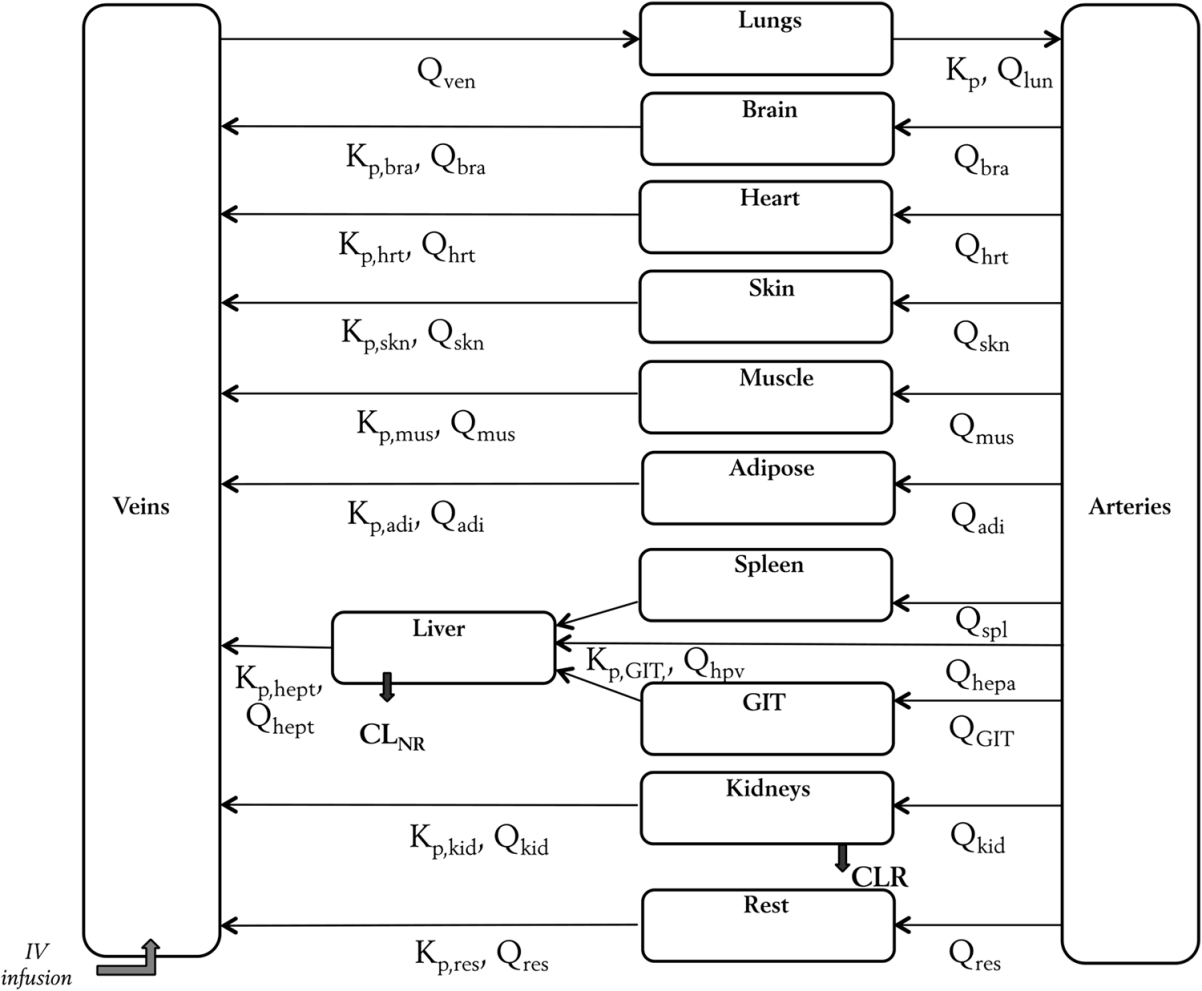
Structure of the WB-PBPK model developed for ciprofloxacin. Volumes of different tissue compartments (*V*) and blood flows to tissues (*Q*) were described on the basis of individual weight and gender

A frequentist prior approach, using the normal-Wishart prior subroutine (NWPRI) in NONMEM, was applied. With this approach, uncertainty in the prior Kp values can be considered in the estimation. An uncertainty of 25 % around the typical prior values was applied for each tissue and implemented as a normal distribution on log-scale [33]. A generic structure of a WB-PBPK model was adopted assuming perfusion limited drug distribution in all tissues (Fig. 1) [34]. Different physiological parameters including blood flow to different organs (Q) as well as organ volumes (V) were taken from the literature [35, 36], and incorporated in the model as a function of weight and gender of the individual patient. Instead of using the typical weight of 70 kg individual patient weights were used to calculate the volume of different tissues as fraction of total body weight. Gender based differences in the body composition were also accounted for when defining the tissue volumes and blood flows (see NONMEM code in online appendix) [35, 36].

Clearance (CL) of ciprofloxacin was divided into renal and non-renal clearance in the WBPBPK model (Eq. 2). For a typical patient with a CRCL of 120 ml min^−1^ approximately 45 % of ciprofloxacin is excreted by the kidneys [37], resulting in a prior mode for non-renal clearance (CL_NR_) of 7.17 l h^−1^ and for fraction secretion (f_secretion_) of 0.57. Individual CRCL values were used to calculate the renal clearance (CL_R_) according to Eq. (3)

2 $$\rm CL \, = \, \rm CL_{R} \, + \, CL_{NR}$$

3 $$\rm CL_{R} \, = \, \rm CRCL \times f_{u,plasma} \times \left( {1 + f_{Secretion} } \right)$$

where f_u,plasma_ is the fraction unbound of ciprofloxacin in plasma (fu = 0.65) [37] and f_Secretion_ is the here estimated fraction of the ciprofloxacin renal clearance that is dependent on secretion from blood to renal tubules. Unexplained inter-individual variability (IIV) was explored without applying a prior.

The differential equations describing the mass transfer in the WBPBPK model are illustrated in the NONMEM code (see online appendix). CL and Kp values were estimated on the log-scale.

### Model predictions

During all predictions the doses of ciprofloxacin was kept at 400 mg b.i.d. and administered intravenously as a 1 h infusion. To predict unbound ciprofloxacin concentration–time profiles in different tissues, the total concentrations of ciprofloxacin predicted by the WB-PBPK model were converted to unbound concentrations by using Eq. (4) where the unbound fraction in extracellular fluid (fue) is defined [38].

4 $$\rm fue \, = \, \rm 1/\left( {1 + E/P \times \left( {1 - fu,plasma} \right)/fu,plasma} \right)$$

E/P is the ratio of albumin in extracellular fluid to plasma. E/P values for each organ or tissue was taken from literature values reported for rats [14].

The final WB-PBPK model was coupled to a PKPD model of ciprofloxacin for *E. coli* [22] to quantitatively predict the time-course of the bacterial killing in the different tissues. The details of the PKPD model have earlier been described and the differential equations are included in the NONMEM code (online appendix). In brief, the model includes parameters for natural growth (1.70 h^−1^) and death (0.179 h^−1^), a structure that allows for characterization of a maximum bacterial concentration, an E_max_-model for the ciprofloxacin-induced rate of killing, the presence of a less susceptible subpopulation (0.819 bacteria per 10^6^ CFU ml^−1^ start inocula), and a 5.34 h time period where the bacteria were susceptible to ciprofloxacin-induced filament formation and hence not countable when plated. The predicted unbound ciprofloxacin concentrations in the extracellular space of the various tissues and organs were in this step used to drive the concentration-dependent rate constant of bacterial killing. The extracellular concentrations were used to drive the effect due to the fact that *E. coli* are assumed to be in the extracellular space. Predictions were made for different *E. coli* strains (LM347, LM202, LM378, LM625, LM693 and LM707) of variable resistance levels (MIC ranging from 0.023 to 48 mg l). When two strains were combined to explore the rate and extent of mutant take-over, each strain had a starting bacteria concentration of 5·10^6^ CFU ml^−1^.

In a next step, the effect of the immune response on the bacterial clearance was added. In accordance to previous publications [19, 20], the killing by neutrophils was here assumed to be dependent on both the neutrophil count and the number of bacteria in the tissue and a term (Eq. 5) for the immune response was added to all bacterial compartments (see online appendix)

5 $$\rm dB/dt \, = \, \rm \ldots - \, Kkill\_ANC \cdot \left( {ANC/\left( {ANC + ANC50} \right)} \right) \cdot \left( {1 - B/\left( {B + B50} \right)} \right) \cdot B .$$

The term in Eq. (5) describes the bacterial kill by the immune system which is dependent on both the number of neutrophils and the bacterial burden. B is the bacterial concentration (CFU ml^−1^), Kkill_ANC describes the maximum rate at which neutrophils can kill the pathogens (1.74 h^−1^), ANC is the absolute neutrophil count (here assumed a constant value of 2500 cells µl^−1^), ANC50 is the number of neutrophils required to achieve 50 % of maximum killing by immune cells (190.8 cells µl^−1^), and B50 is concentration of bacteria required to saturate the immune cell response by 50 % (430 × 10^4^ CFU ml^−1^). Using this model, the bacterial killing by the immune cells will be according to a first order process (according to Kkill_ANC) at low bacterial concentrations. However, when approaching higher bacterial concentrations saturation will occur and the killing will approach a zero order process (constant killing rate). The immune response was assumed to affect all bacteria similarly, regardless of the susceptibility and growth state of the bacteria (see online appendix).

### Software

Perl speaks NONMEM (PsN) was used to facilitate execution, to summarize results and to evaluate the models. A more complex model was selected when the difference in the Objective Function Value reported by NONMEM reduced at least 10.83 units (p < 0.001, for one degree of freedom). To evaluate the typical trends predicted by the model, as well as the model’s ability to describe the variability, prediction corrected Visual predictive checks (VPCs) were performed. Piraña, Xpose and R (ggplot) were also used for plotting and analyzing the results from different models and predictions [39–42].

## Results

### Development of the WB-PBPK model for ciprofloxacin

**Fig. 2:**
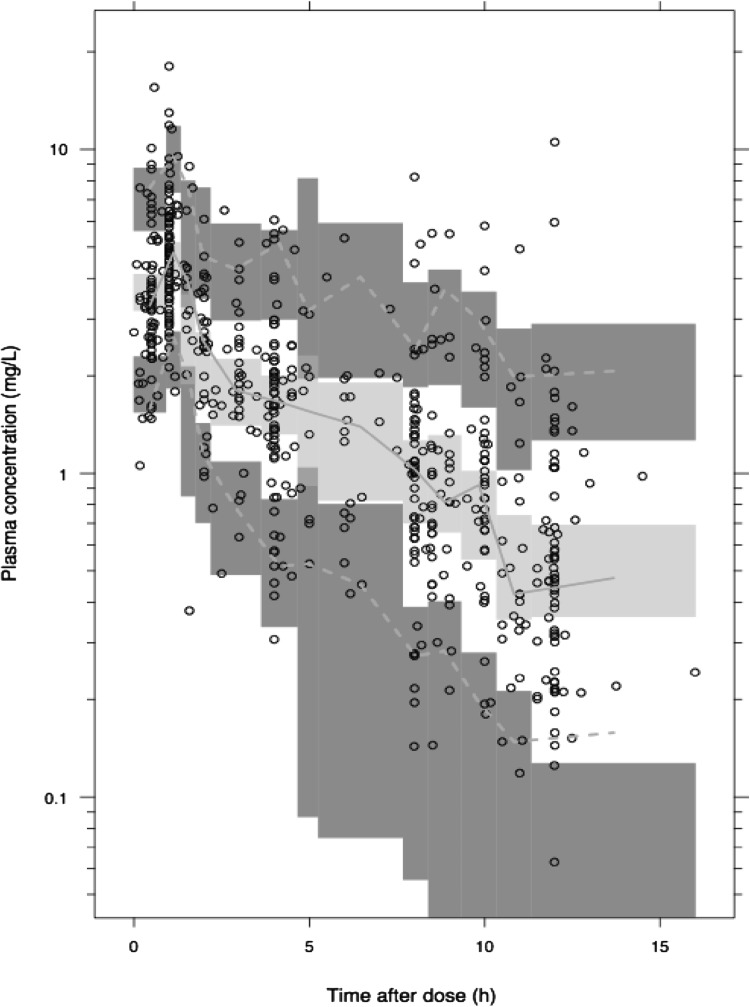
Visual predictive check for the WB-PBPK model.* Dotted red lines* are the median and the 5th and 95th percentiles of the observed plasma-concentration data.* Shaded areas* represent the 95 % confidence interval around the median and the 5th and 95th percentiles based on simulations from the model (n = 500) (Color figure online)

A WB-PBPK model characterizing both the typical trends and variability of the available ciprofloxacin plasma concentration data was successfully developed as illustrated in the VPC (Fig. 2). Inclusion of the clinical Kp values as priors was needed to stabilize the WB-PBPK model and the parameter estimates including clearance and tissue Kp values were comparable to the literature values (Table 1). The estimate for the Kp value in muscle deviate the most (except for “Rest”) and was estimated to be 60 % higher than the literature prior value. The renal clearance was for a typical patient with a CRCL of 110 ml min^−1^, estimated to be 49 % of the total clearance, while for a patient with a CRCL of 50 ml min^−1^ renal clearance was predicted to constitute 13 % of the total clearance. The final model included IIV for clearance and for Kp values (a common value for all).

**Table 1:** Prior values applied based on clinical data from the literature and the here estimated parameters of the developed WB-PBPK model

	Prior values normal scale (uncertainty)	Model estimate log-scale (±SE)	Model estimate normal scale (RSE)
CL_NR_ (l h^−1^)	7.17 (25 %)	2.60 ± 0.14	13.5
f_secretion_	0.57 (25 %)		0.674 (26 %)
Kp,lung	3.3 (25 %)	1.20 ± 0.25	3.32
Kp,brain	0.771 (25 %)	−0.257 ± 0.25	0.773
Kp,heart	3.67 (25 %)	1.30 ± 0.25	3.67
Kp,skin	0.718 (25 %)	−0.335 ± 0.24	0.715
Kp,muscle	1.6 (25 %)	−0.0229 ± 0.16	0.977
Kp,adipose	0.449 (25 %)	−0.885 ± 0.23	0.413
Kp,spleen	1.954 (25 %)	0.668 ± 0.25	1.95
Kp,GIT	3.39 (25 %)	1.21 ± 0.23	3.35
Kp,liver	3.67 (25 %)	1.27 ± 0.23	3.56
Kp,kidney	8.2 (25 %)	2.09 ± 0.25	8.09
Kp,rest	2.77 (25 %)	1.35 ± 0.11	3.86
IIV CL (CV %)	–		56 (9.3 %)
IIV Kp (CV %)	–		55 (15 %)
Proportional residual error (%)	–		33 (7.1 %)

f_secretion_ and CL_NR_ represent the fraction of ciprofloxacin renal clearance which relies on secretion into renal tubules and non-renal clearance, respectively

### Model predictions of unbound concentrations

**Fig. 3:**
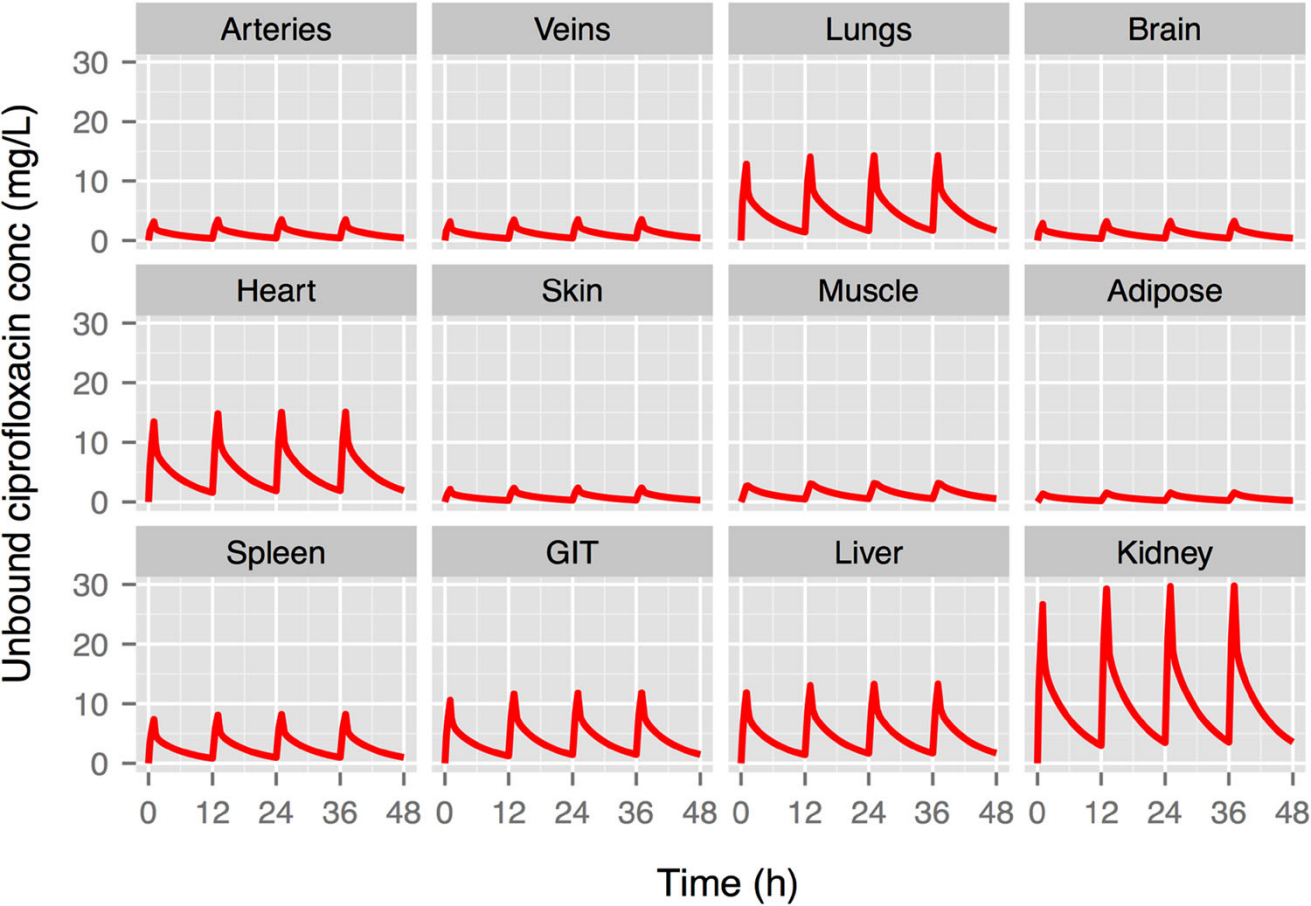
Predicted extracellular tissue concentration–time profiles of unbound ciprofloxacin following a dosing regimen of 400 mg b.i.d

Profiles of unbound ciprofloxacin concentration in different tissues were predicted from the developed WB-PBPK model (Fig. 3). Kidney and lung were predicted to have higher exposures as compared to the other organs while muscle, brain and adipose were predicted to have relatively low exposures to ciprofloxacin. Cmax was achieved at the end of the 1 h constant rate infusion in plasma as well as in all other tissues and organs, depicting the rapid distribution of ciprofloxacin.

### Combining the WBPBPK with the PKPD model

**Fig. 4:**
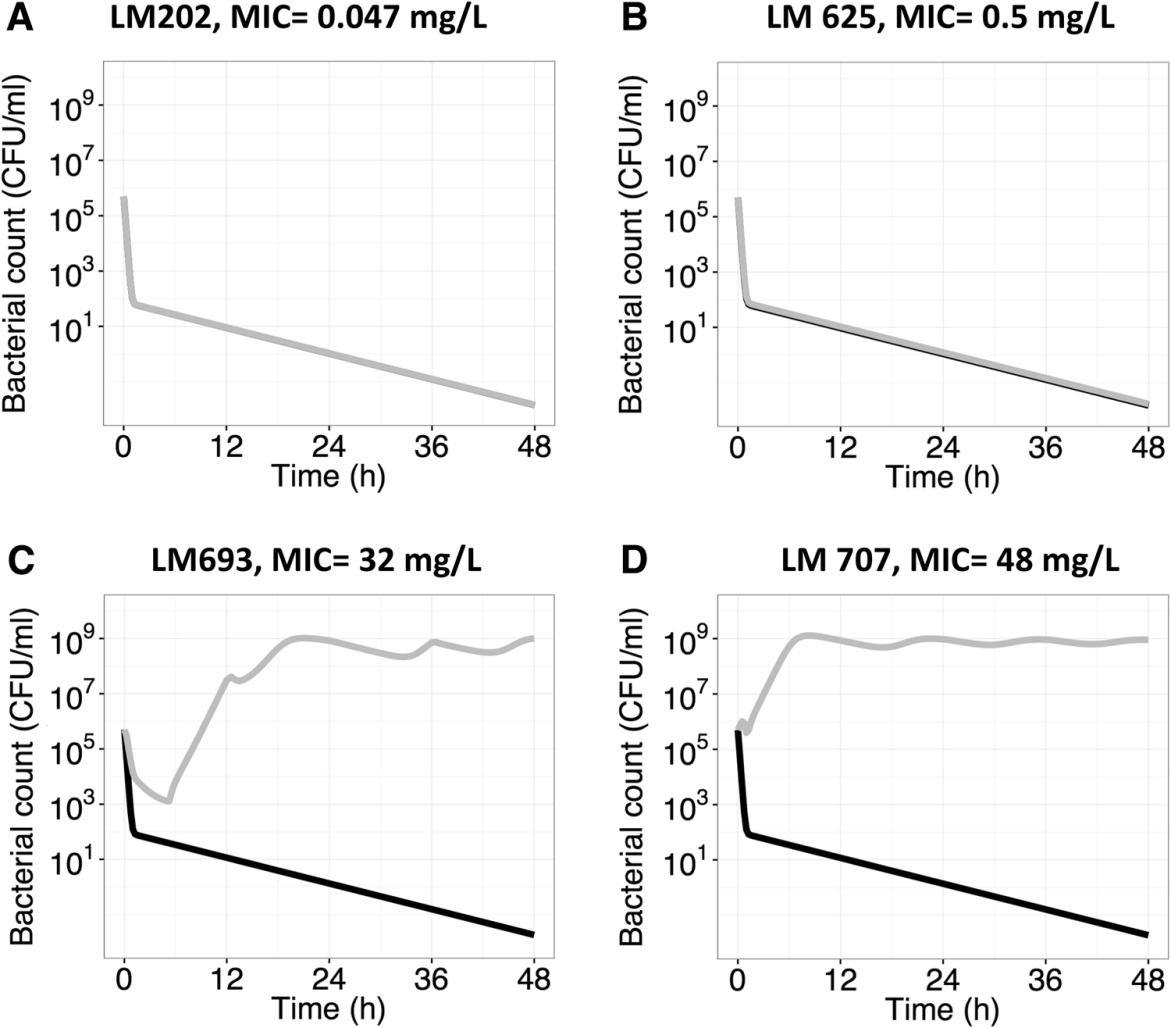
Predictions of the time course of bacterial killing of different *E. coli* strains in the extracellular compartment of kidney following a ciprofloxacin dose of 400 mg b.i.d. *Grey lines* represents the resistant bacterial strain while *black lines* represent wild type bacterial strain (LM347, MIC = 0.023 mg l^−1^). Please note that in A the two strains are overlapping

When the predicted PK profile of unbound ciprofloxacin from the WB-PBPK model was driving the PKPD model, the rate and extent of take-over of mutant bacteria in different tissues could be predicted and compared. For kidney, representing a scenario of pyelonephritis, the 400 mg b.i.d. dosing regimen was predicted to be high enough to clear the bacterial population of not only wild type but also mutants of *E. coli* with intermediate level of resistance (i.e. MIC < 10 mg l^−1^) (Fig. 4a, b). However the highly resistant strains (MIC > 25 mg l^−1^) were not killed off despite the high ciprofloxacin exposures in kidney (Fig. 4c, d).

**Fig. 5:**
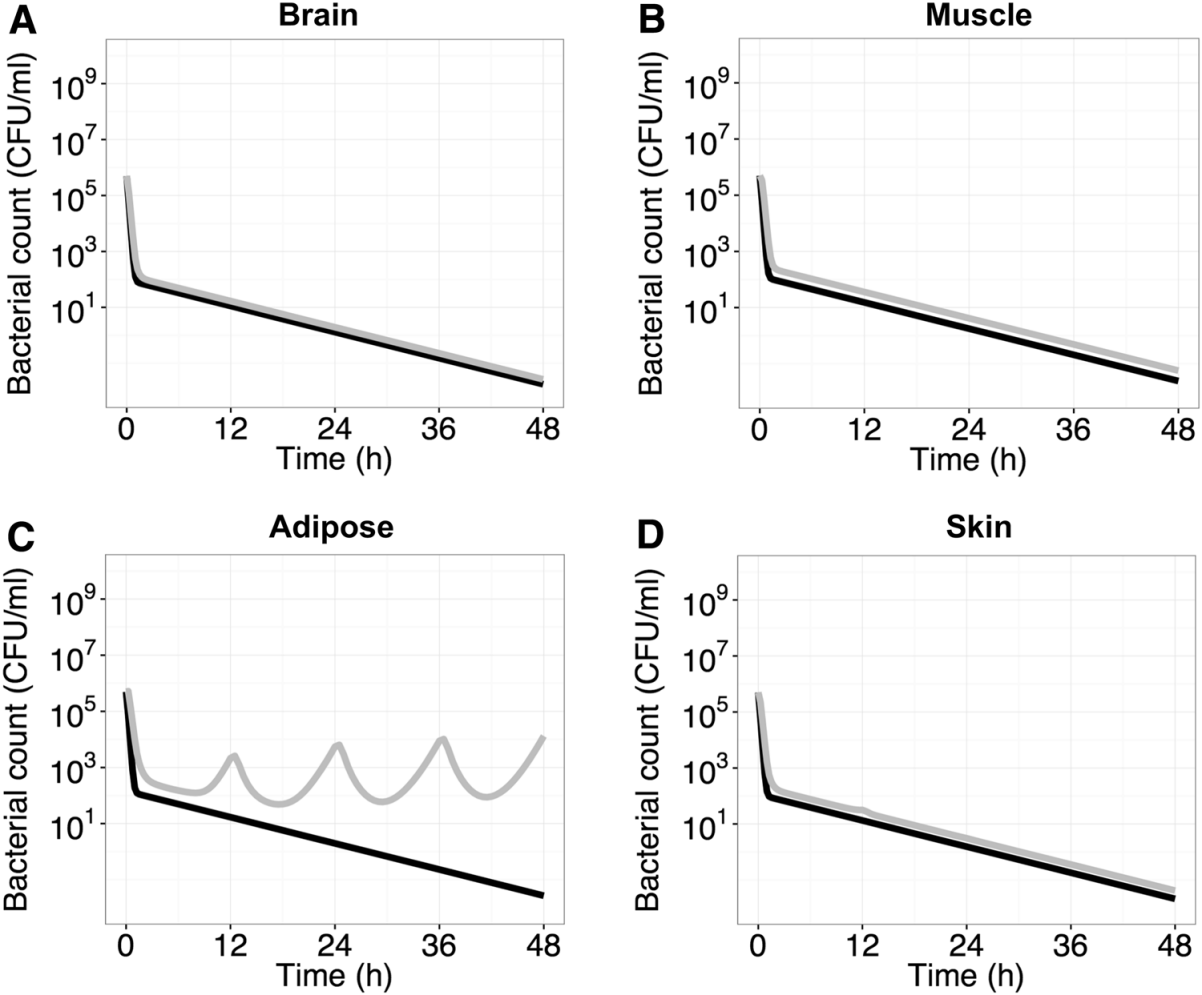
Predictions of the time course of bacterial killing of *E. coli* strains LM347 (*black*) and LM625 (*grey*) in the extracellular compartment of different tissues (brain, muscle, adipose and skin) following a ciprofloxacin dose of 400 mg b.i.d

Similarly, when the bacteria time-course of the *E. coli* strain LM625 (MIC = 0.50 mg l^−1^) was predicted with the different tissue exposures the bacteria was eliminated from the brain, muscle and skin while re-growth was evident in adipose tissue (Fig. 5).

### Addition of neutrophil effect on WBPBPK-PKPD model

**Fig. 6:**
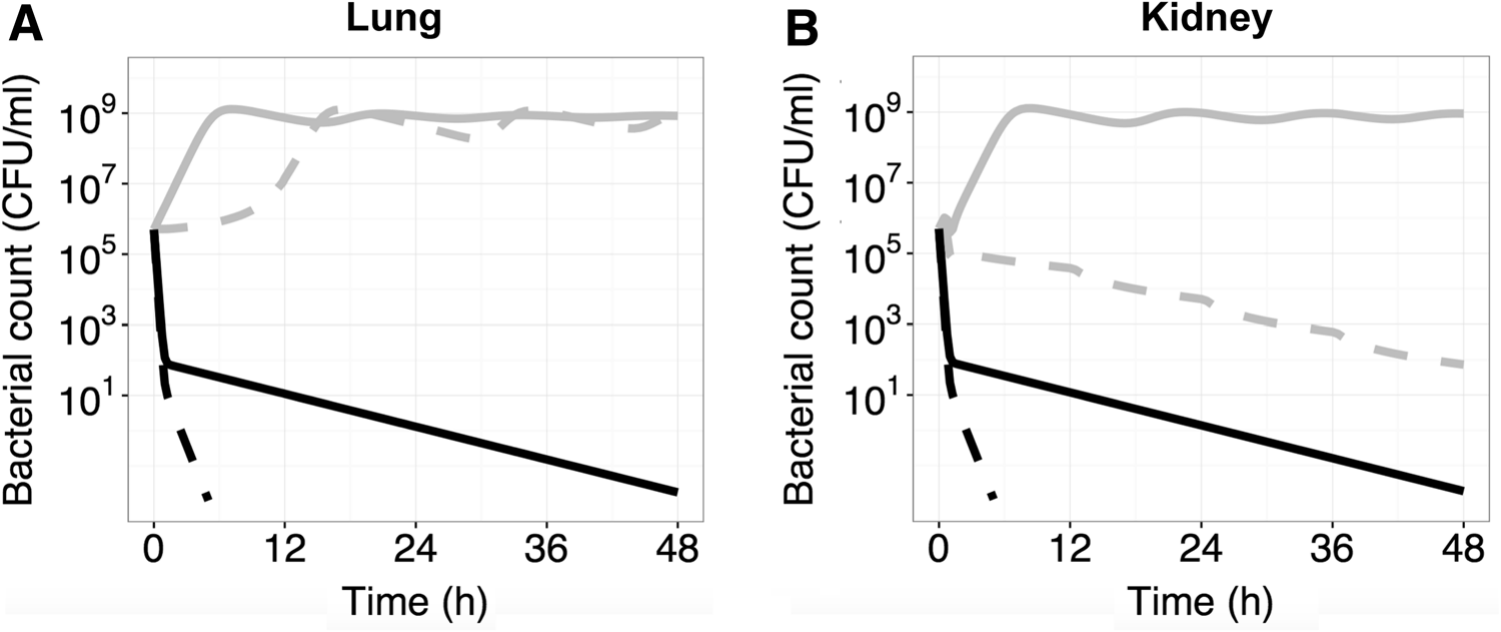
Predictions of the time course of bacterial killing of *E. coli* strains LM347 (*black*) and LM707 (*grey*) in lung and kidney following administration of ciprofloxacin 400 mg b.i.d. with (*dashed lines*) and without (*solid lines*) addition of function for immune response

Lastly the effect of neutrophils was added to the model and predictions were made for the wild type and the most resistant *E. coli* strain, LM707 (MIC = 48 mg l^−1^) in lung and kidney. For the wild-type bacteria, it was predicted that the combination of high exposures of ciprofloxacin and bacterial killing by neutrophils will quickly result in total bacterial eradication. For the resistant strain, the combination was predicted to eliminate the resistant bacteria from kidney in contrast to ciprofloxacin exposure alone (Fig. 6a). In lung, which had the second highest exposure of ciprofloxacin relative to kidney, even adding the effect of immune response was not enough to eliminate the resistant strain and the model predicted re-growth (Fig. 6b).

## Discussion

A WB-PBPK model was successfully developed based on plasma concentration data of ICU patients. The model did not only capture the trends in the observed data but also described the variability in the data as shown in the VPC (Fig. 2). By using clinical Kp values for different tissues as priors a full WB-PBPK model could be developed based on plasma concentration data only. A similar strategy has also been successfully applied for CMS and colistin [43].

This work is the first example of a WB-PBPK model for ciprofloxacin which was implemented using non-linear mixed effects modeling and frequentist priors ($PRIOR in NONMEM). Using informative priors on clinical Kp values, and information on blood flows and tissue volumes from the literature, the full WB-PBPK model could weigh the individual information from the available plasma concentration data to characterize the PK in the study population. Using priors is a good way to provide supportive information from earlier studies and to stabilize a WB-PBPK model in NONMEM [33].

Estimated Kp values were in good agreement with the priors (Table 1). There are some notable variations; e.g. muscle, which is a relatively big tissue compartment, had a 60 % higher Kp estimate as compared to what has been reported in the literature. One reason could be the relatively bigger size of muscle in comparison to other tissues, and a deviation from the prior can thereby have a clear impact on the PK profile. Kp values for relatively small organs e.g. spleen and pancreas were unchanged from the prior, reflecting that either there is not enough information in the data to drive them away from the prior values or estimates and observed values are well in agreement. Also the estimated lung and kidney Kp values relies primarily on the experimentally determined Kp values rather than on the plasma concentration data since the deviations from the prior was small, and the uncertainty was the same. Furthermore, there have been some indications of involvement of active efflux transporters, e.g. P-gp and BCRP, on ciprofloxacin distribution in different tissues. There is however limited evidence of any significant impact of these active transporters in vivo for ciprofloxacin [44, 45]. Due to lack of specific data the current model does not account for active processes and perfusion limited drug distribution was assumed in all tissues. Renal clearance was dependent on the kidney function and with decreased CRCL of 50 ml min^−1^ renal clearance was reduced to 16 % of total clearance.

Based on the developed model, the concentration time profile of ciprofloxacin was predicted in different tissues. The highest unbound concentrations were observed in kidney and lung, which is in line with the ciprofloxacin treatment indications of pneumonia and urinary tract infections. The NONMEM implementation allowed for connecting the WB-PBPK model to an in vitro based PKPD model, where the predicted unbound concentration in extracellular fluid was driving the bacterial killing. It was also illustrated how a function for the immune response could be added to investigate the impact on the rate and extent of bacterial killing.

During recent years, the use of PBPK models has become an integral tool in drug development. Here we suggest a framework in which a WB-PBPK model is combined with a PKPD model that can describe the time-course of bacterial growth and kill. Thereby the relative kill of different dosing regimens and bacterial strains can be compared for different tissues. In the in vitro PKPD model drug concentration in the test tube was used to drive the bacterial killing while here the predicted unbound drug concentration–time profile in different tissues was replacing the static test tube concentration in the E_max_-model. Similar predictions based on unbound plasma concentrations in plasma have earlier been used to support treatment regimens of e.g. gentamicin, colistin and meropenem [46].

It should be noted that there are several assumptions in the predictions illustrated. For example, the predictions assume that in vivo bacterial growth and killing with the antibacterial will be similar to the in vitro test tube situation. This work is however one step closer to the in vivo situation compared to the test tube situation as we use the predicted time course of unbound ciprofloxacin at potential infection sites based on clinical data. The predicted concentration–time profiles are most likely a better representation of the concentration in the extracellular tissue fluids than the plasma concentration time-profiles that are typically used to illustrate bacterial killing in in vitro kinetic systems. This modeling framework illustrates a way of combining PK and PKPD information from in vitro, in vivo and clinical data to make a best guess. Once the separate model components have been developed and connected, different scenarios can be simulated and different hypothesis be tested. For example, the framework could be applied for comparing dosing regimens between different study populations with different PK, and different types of bacteria and/or infection sites. The model could also be used to explore the potential for new indications, i.e. different infection sites where the drug concentrations are predicted to be sufficiently high for a good bactericidal effect. Here we explored the bacterial killing using different bacterial strains with different degree of drug susceptibility, but also the impact of e.g. a lower fitness or growth rate in the tissues could be tested.

The immune response is an important factor for the outcome of an antibacterial therapy. An extreme example of the complications with immunodeficiency is HIV patients who require higher doses and combination antibiotic treatment as compared to an immunocompetent patients [47, 48]. Most often the efficacy of antibacterials is estimated based on the data from in vitro time kill curve experiments and/or in vivo studies using neutropenic mice, thus ignoring the role of neutrophils and other immune cells. Recently, Lyons et al. described a physiologically based PKPD model of rifampin therapy in a mouse tuberculosis infection model also accounting for dynamics for host immune response to *Mycobacterium tuberculosis* infection [49]. In this work we wanted to also illustrate how this type of framework can be used to explore the relative response of the immune system and drug treatment. With the addition of immune response, the highly resistant strain, LM707, was predicted to be eliminated from kidney while in lung, which had slightly lower levels of ciprofloxacin, addition of an immune response was not enough to eliminate the bacteria (Fig. 6b). The equation applied for the immune response was based on two in vivo studies quantifying the effect of neutrophils and bacterial burden (Eq. 5). It should be noted that these predictions are based on the effect as quantified in mice experiments and that further studies to elucidate the quantitative impact of the immune cells are warranted.

In this project we suggest a framework where data from different sources and different models are integrated to simulate the time course of unbound concentration and bacterial killing at the site of drug action. This approach represents a possibility to predict the therapy outcome in different patient populations by combining the information from different sources. In its present state this framework is based on strong assumptions as, for example, there is limited information available on possible differences in bacterial growth rates and inoculum sizes at different infection sites, and data on the impact of the immune system is sparse. Despite its limitations it represents a show case and a step in the direction of model-informed drug discovery and development (MID3) [11] by incorporating information from several sources and the application of translational approaches. This type of WB-PBPKPD modeling framework has thereby the potential to help guiding drug development of antimicrobials. The approach can especially be valuable given that we are rapidly facing an era of increasing resistance, where there is an urgent need for new antimicrobial agents and effective drug combinations. Given the parameterization of the WB-PBPK model into volumes and flows, the framework can also be extended and adapted to other patient populations where differences in drug disposition is expected, such as for pediatric and septic shock patients.

## Conclusion

The developed WB-PBPK model successfully described the plasma concentration profile data from patients by characterizing both typical trends and variability where parameter estimation was based on plasma concentrations combined with prior information on ciprofloxacin Kp values and clearance. Linking the predicted unbound extracellular concentration–time profiles from the WB-PBPK model with a PKPD model developed based on in vitro data is a promising approach for predicting infection site specific time courses of bacterial killing. This type of framework can be applied to support treatment strategies for selection of antibiotics for different indications and investigate the impact of different levels of drug susceptibility for the bacteria.

## Electronic supplementary material

Below is the link to the electronic supplementary material.

Supplementary material 1 (PDF 22 kb)
