# Supplementary material for: A whole-body physiologically based pharmacokinetic (WB-PBPK) model of ciprofloxacin: a step towards predicting bacterial killing at sites of infection
Source: J Pharmacokinet Pharmacodyn. 2016 Aug 30;44(2):69–79. doi: 10.1007/s10928-016-9486-9 (PMC5376394; doi:10.1007/s10928-016-9486-9)
Supplement: Supplementary file 1 — Supplementary material 1 (PDF 22 kb) [file 10928_2016_9486_MOESM1_ESM.pdf]

```
;; 1. Based on: run17
;; 2. Description: run16 with PKPD from JAC 2016


$PROB  WB-PBPK-PD-Immune predictions

$INPUT ID TIME AMT TINF RATE CONC DV CMT MDV EVID PER VD SEX AGE WT MDRD COCK
CRCL PROT CM3 IGS2 HB BUN HTE

$DATA cipro_data_ln4.csv IGNORE=# IGNORE=(ID.LT.144)

$SUBROUTINES  ADVAN13 TOL=9

$PRIOR NWPRI NTHETA=32 NETA=2 NTHP=13 NETP=0 NPEXP=1

$MODEL

;;----------------------------PBPK Compartments---------------------------;;
COMP = (ART)                                            ; 1- Arteries
COMP = (VEN)                                            ; 2- Veins
COMP = (LUN)                                            ; 3- Lungs
COMP = (BRA)                                            ; 4- Brain
COMP = (HRT)                                            ; 5- Heart
COMP = (SKN)                                            ; 6- Skin
COMP = (MUS)                                            ; 7- Muscle
COMP = (ADI)                                            ; 8- Adipose
COMP = (SPL)                                            ; 9- Spleen
COMP = (GIO)                                            ; 10-GIO
COMP = (LIV)                                            ; 11-Liver
COMP = (KID)                                            ; 12-Kidneys
COMP = (RES)                                            ; 13-Rest
COMP = (ELI)                                            ; 14-Eliminated drug

;;----------------------------PKPD Compartments---------------------------;;
;; --------WILD TYPE
COMPARTMENT=(S)               ; 15-Compartment S, suspectible
COMPARTMENT=(R)               ; 16-Compartment R, resting

COMPARTMENT=(SPE)             ; 17-Compartment SPE, Pre-existing resistant
bacteria
COMPARTMENT=(NP)              ; 18-Compartment NP, non-plateable (non-colony
forming)
COMPARTMENT=(RPE)             ; 19-Compartment R, resting Pre-existing
resistant bacteria
COMPARTMENT=(NPPE)            ; 20-Compartment non-plateable (non-colony)
forming Pre-existing resistant bacteria

;; --------Resistant bacteria

COMPARTMENT=(S1)               ; 21-Compartment S, suspectible
COMPARTMENT=(R1)               ; 22-Compartment R, resting

COMPARTMENT=(SPE1)             ; 23-Compartment SPE, Pre-existing resistant
bacteria
COMPARTMENT=(NP1)              ; 24-Compartment NP, non-plateable (non-colony
forming)
COMPARTMENT=(RPE1)             ; 25-Compartment R, resting Pre-existing
resistant bacteria
COMPARTMENT=(NPPE1)            ; 26-Compartment non-plateable (non-colony
forming) Pre-existing resistant bacteria

$PK
IF(NEWIND.NE.2)DOSE=AMT
```

```
;;---------------------------PBPK-------------------------------------;;
;; ------------------------ Tissue volumes (L) ------------------------- ;;
IF (SEX.EQ.0) THEN
VART = 0.017083*(WT)                                    ; Arterial volume (L)
VVEN = 0.051248*(WT)                                    ; Venous volume (L)
VLUN = 0.00643836*(WT)                                  ; Lungs weight (Kg) ICRP
VBRA = 0.0191781*(WT)                                   ; Brain volume (L)
VHRT = 0.004167*(WT)                                    ; Heart volume (L) ICRP
VSKN = 0.0383*(WT/1.18)                                 ; Skin volume (L)
VMUS = 0.2916*(WT)                                      ; Muscle volume (L) ICRP
VADI = 0.3*(WT/0.916)                                   ; Adipose volume (L) ICRP
VSPL = 0.00247*(WT)                                     ; Spleen volume (L) ICRP
VGIO = 0.01644*(WT)                                     ; GIO volume (L)
stomach+SI colon ICRP
VHEP = 0.020724*(WT)                                    ; Hepatic volume (L)  ICRP
VKID = 0.0042466*(WT)                                   ; Kidney volume (L) ICRP
VRES = 0.23220194*(WT)                                  ; Rest of body volume
bones ICRP
ENDIF

IF (SEX.EQ.1) THEN
VART = 0.01918*(WT)                                      ; Arterial volume (L)
VVEN = 0.05753*(WT)                                      ; Venous volume (L)
VLUN = 0.00643836*(WT)                                   ; Lungs weight (Kg) ICRP
VBRA = 0.01918*(WT)                                      ; Brain volume (L)
VHRT = 0.004521*(WT)                                     ; Heart volume (L) ICRP
VSKN = 0.045205*(WT/1.18)                                ; Skin volume (L)
VMUS = 0.3973*(WT)                                       ; Muscle volume (L) ICRP
VADI = 0.171233*(WT/0.916)                               ; Adipose volume (L) ICRP
VSPL = 0.00247*(WT)                                      ; Spleen volume (L) ICRP
VGIO = 0.01644*(WT)                                      ; GIO volume (L)
stomach+SI colon ICRP
VHEP = 0.02466*(WT)                                      ; Hepatic volume (L)  ICRP
VKID = 0.004247*(WT)                                     ; Kidney volume (L) ICRP
VRES = 0.23612164*(WT)                                   ; Rest of body volume
bones ICRP
ENDIF

;; -------------------------- Blood flows (L/H) ------------------------ ;;

CO  = (15*(WT)**(0.74))                                  ; Cardiac output in L/h


QART = CO                                                ; Artery Blood flow
QVEN = CO                                                ; Venous blood flow
QLUN = CO                                                ; Lung blood flow

IF (SEX.EQ.0) THEN
QBRA = CO*(0.12)                                         ; Brain blood flow
QHRT = CO*(0.05)                                         ; Heart blood flow
QSKN = CO*(0.05)                                         ; Skin blood flow
QMUS = CO*(0.12)                                         ; Muscle blood flow
QADI = CO*(0.085)                                        ; Adipose blood flow
QSPL = CO*(0.03)                                         ; Spleen blood flow
QGIO = CO*(0.16)                                         ; GIO blood flow
QKID = CO*(0.17)                                         ; Kidney blood flow
QHEPA= CO*(0.065)                                        ; Hepatic arterial blood
flow
QRES = CO*(0.15)                                         ; Rest of Body blood flow
ENDIF

IF (SEX.EQ.1) THEN
QBRA = CO*(0.12)                                         ; Brain blood flow
```

```
QHRT = CO*(0.04)                                       ; Heart blood flow
QSKN = CO*(0.05)                                       ; Skin blood flow
QMUS = CO*(0.17)                                       ; Muscle blood flow
QADI = CO*(0.05)                                       ; Adipose blood flow
QSPL = CO*(0.03)                                       ; Spleen blood flow
QGIO = CO*(0.15)                                       ; GIO blood flow
QKID = CO*(0.19)                                       ; Kidney blood flow
QHEPA= CO*(0.065)                                      ; Hepatic arterial blood
flow
QRES = CO*(0.135)                                      ; Rest of Body blood flow

ENDIF
QHEPT= QHEPA + QSPL + QGIO                             ; Total hepatic blood
flow HEPA+SPL+GIO)


;; ---------------------------- Unbound fraction ------------------------------
;;

FUP = 0.65                                             ;

;; --------------------------- Clearance parameters ----------------------------
;;

TVCLH= EXP(THETA(1))                                   ; Hepatic or Non-renal CL
CLH  = TVCLH*EXP(ETA(1))

TVRSEC = EXP(THETA(2))                                 ; Factor renal secretion
RSEC = TVRSEC

CRCL2=CRCL
IF (CRCL.GT.150) CRCL2=150                             ; Cap high values where
Cockcroft-Gault less reliable
CLR   = (((CRCL2*60/1000)*FUP)*(1+RSEC))*EXP(ETA(1))
CL    = CLR+CLH


;; -------------------------- Partition Coefficients ---------------------------
;;

KLUN = EXP(THETA(3))*EXP(ETA(2))
KBRA = EXP(THETA(4))*EXP(ETA(2))
KHRT = EXP(THETA(5))*EXP(ETA(2))
KSKN = EXP(THETA(6))*EXP(ETA(2))
KMUS = EXP(THETA(7))*EXP(ETA(2))
KADI = EXP(THETA(8))*EXP(ETA(2))
KSPL = EXP(THETA(9))*EXP(ETA(2))
KGIO = EXP(THETA(10))*EXP(ETA(2))
KHEP = EXP(THETA(11))*EXP(ETA(2))
KKID = EXP(THETA(12))*EXP(ETA(2))
KRES = EXP(THETA(13))*EXP(ETA(2))

IF (TIME.EQ.0) OCUMDOSE = 0
IF (AMT.GT.0) THEN
TDOS=TIME
TAD=0.0
CUMDOSE = OCUMDOSE + AMT
ENDIF
OCUMDOSE = CUMDOSE
IF (AMT.EQ.0) TAD=TIME-TDOS

;;-----------------------------PK(PD) ------------------------------------------
-;;
```

```
;;-- growth rate
KGS    = THETA(14)                  ;;Typical value growth rate
NONUSE   = THETA(15);

;;-- death rate
KK    = 0.179   ;;Typical value death rate constant, FIXED from Nielsen et al
2007

;;-- drug EMAX

EMAX  = THETA(16)

;;-- drug EC50
EC50 = THETA(17)             ;; EC50  WT
EC501 = THETA(18)            ;; EC50 Resistant strain
;;-- hill factor
GAM  = THETA(19)

NONUSE2 = THETA(20)

;; -- feed
PC  = THETA(21)*0.0000001    ;; proportionality constant;;THETA scalled,
;;;;                         ;; corresponds to maximum bacterial load, around
10^9, see equations
SBASE=500000                 ;; Start inocculum
SBASE1=500000                ;; Inoculum for resistant strain
;;--growth rate 2
KGS2 = THETA(22)             ;; Growth rate constant pre-existing resistant
strain
NONUSE3 = THETA(23)

;;-- drug EC502
EC502= THETA(24)           ;; EC50 pre-existing resistant strain
NONUSE4 = THETA(25)

;;--pre-existing resistant at start
MUT= THETA(26)
NONUSE5  = THETA(27)

;;--compartment initialization
A_0(15)= SBASE*(1-MUT*0.000001)            ;compartment initialization Growing
(S)
A_0(16)= 0                                 ;Start conc bacteria in resting
condition

A_0(17)= MUT*0.000001*SBASE                ;Start pre-exisiting resistant
A_0(18)= 0                                 ;Start non-colony forming
A_0(19)= 0                                 ;Start pre-exisiting resistant
resting
A_0(20)= 0                                 ;Start pre-exisiting resistant non-
colony forming

;;----------------Resistant strain
A_0(21)= SBASE1*(1-MUT*0.000001)           ;compartment initialization IBASE
A_0(22)= 0                                 ;Start amount cells in resting
condition

A_0(23)= MUT*0.000001*SBASE1               ;Start mutant strain pre-exisiting
resistant Growing (S)
A_0(24)= 0                                 ;Start mutant strain non-colony
forming
A_0(25)= 0                                 ;Start mutant strain pre-exisiting
resistant resting
```

```
A_0(26)= 0                                 ;Start mutant strain pre-exisiting
resistant non-colony forming

;;-- K non-colony forming compartment

KSNP1    =  THETA(28)

KNPS2    =  THETA(30)


;;-- MTIME - shuts off KNP1--time for non platable formation to stop
MTIME(1) = 0
MTIME(2) = MTIME(1)+THETA(31)

$DES
;; ------------------------- Compartment concentrations -----------------------
;;

C1 = A(1)/VART                                        ; AMT/Arterial volume
C2 = A(2)/VVEN                                        ; AMT/Venous volume
C3 = A(3)/VLUN                                        ; AMT/Lung volume
C4 = A(4)/VBRA                                        ; AMT/Brain volume
C5 = A(5)/VHRT                                        ; AMT/Heart volume
C6 = A(6)/VSKN                                        ; AMT/Skin volume
C7 = A(7)/VMUS                                        ; AMT/Muscle volume
C8 = A(8)/VADI                                        ; AMT/Adipose volume
C9 = A(9)/VSPL                                        ; AMT/Spleen volume
C10= A(10)/VGIO                                       ; AMT/GIO volume
C11= A(11)/VHEP                                       ; AMT/Hepatic volume
C12= A(12)/VKID                                       ; AMT/Kidney volume
C13= A(13)/VRES                                       ; AMT/Rest volume

;; ------------------------------ Abbreviations ------------------------------
;;


VENIN1 = (C4*QBRA/KBRA)+(C5*QHRT/KHRT)+(C6*QSKN/KSKN)+(C7*QMUS/KMUS)
VENIN2 = (C8*QADI/KADI)+(C11*QHEPT/KHEP)+(C12*QKID/KKID)+(C13*QRES/KRES)
VENOUT = (QLUN*C2)

HEPIN  = (C1*QHEPA)+(C9*QSPL/KSPL)+(C10*QGIO/KGIO)
HEPOUT = (C11*QHEPT/KHEP)+(C11*CLH*FUP/KHEP)
TOT    = A(1)+A(2)+A(3)+A(4)+A(5)+A(6)+A(7)+A(8)+A(9)+A(10)+A(11)+A(12)+A(13)
TOTA   = TOT + A(14)

; ---------------------------------(PK)PD-------------------------------------
;;

; -------------------------------Drug Conc----------------------------------;;
CAB = C3*0.79 ; EXAMPLE Lung

; ---------------------------------WT strain----------------------------------
---;;

KV1 =  (CAB/EC50)**THETA(32)/((CAB/EC50)**THETA(32)+THETA(29)**THETA(32))
; conc-driven to nc-compartment
KV11 = (CAB/EC502)**THETA(32)/((CAB/EC502)**THETA(32)+THETA(29)**THETA(32))
; pre-existing mutant
KV2 = EC50/(CAB+0.0000000001)        ; conc-driven from nc-compartment
KV22 = EC502/(CAB+0.0000000001)      ; pre-existing mutant

; ---------------------------------Resistant strain---------------------------
----------;;
```

```
KV110 = (CAB/EC501)**THETA(32)/((CAB/EC501)**THETA(32)+THETA(29)**THETA(32))
KV111 = (CAB/EC502)**THETA(32)/((CAB/EC502)**THETA(32)+THETA(29)**THETA(32))
KV21 =  EC501/(CAB+0.0000000001)
KV221 = EC502/(CAB+0.0000000001)

ANC  = 2500000
KKIL = 1.743
B50  = 4300000
POPMAX = 1800000000
ANC50 = 190800

;;-- see MTIME
FLAG=MPAST(1)-MPAST(2)

;;-- Conversion rate between active/resting cell dependent on cell number
BTOT = A(15)+A(16)+A(17)+A(18)+A(19)+A(20)+A(21)+A(22)+A(23)+A(24)+A(25)+A(26)
SR=PC*BTOT
SR2=SR
RS=0
RS2=0

;EMAX equation for drug effect

DRUGS=0
DRUGS2=0
DRUGS1=0
DRUGS21=0

IF(CAB.GT.0.00000000001)THEN
DRUGS = EMAX*(CAB)**GAM/(EC50**GAM+(CAB)**GAM)      ;; Drug effect sensitive 347
DRUGS2 = EMAX*(CAB)**GAM/(EC502**GAM+(CAB)**GAM)    ;; Drug effect resistant 347
DRUGS1 = EMAX*(CAB)**GAM/(EC501**GAM+(CAB)**GAM)    ;;Drug effect sensitive 707
DRUGS21 =EMAX*(CAB)**GAM/(EC502**GAM+(CAB)**GAM)    ;;Drug effect resistant 707
ENDIF

;; -------------------------- Differential equations --------------------------
;;
;;----------------------------------PBPK-----------------------------------------
;;
DADT(1) = (QLUN*C3/KLUN)-(QART*C1)
DADT(2) = VENIN1+VENIN2-VENOUT
DADT(3) = (QVEN*C2)-(QLUN*C3/KLUN)
DADT(4) = (QBRA*C1)-(QBRA*C4/KBRA)
DADT(5) = (QHRT*C1)-(QHRT*C5/KHRT)
DADT(6) = (QSKN*C1)-(QSKN*C6/KSKN)
DADT(7) = (QMUS*C1)-(QMUS*C7/KMUS)
DADT(8) = (QADI*C1)-(QADI*C8/KADI)
DADT(9) = (QSPL*C1)-(QSPL*C9/KSPL)
DADT(10)= (QGIO*C1)-(QGIO*C10/KGIO)
DADT(11)= HEPIN-HEPOUT
DADT(12)= (QKID*C1)-(QKID*C12/KKID)-C1*CLR
DADT(13)= (QRES*C1)-(QRES*C13/KRES)
DADT(14)= C1*CLR+(C11*CLH*FUP/KHEP)
;; ----------------------------------(PK)PD WILDTYPE----------------------------
---------;;
DADT(15)= KGS*(A(15))-(KK+DRUGS)*(A(15)) - SR*(A(15)) +(KNPS2*KV2)*A(18) -
(KSNP1*KV1)*A(15)*FLAG - KKIL*(ANC/(ANC+ANC50))*(1-A(15)/(A(15)+B50))*A(15)
     ; S, suspectible
DADT(16)=-KK*(A(16)) + SR*(A(15)) - KKIL*(ANC/(ANC+ANC50))*(1-
A(16)/(A(16)+B50))*A(16)             ; R, resting

DADT(17)= KGS2*(A(17))-(KK+DRUGS2)*(A(17)) - SR2*(A(17)) +(KNPS2*KV22)*A(20) -
(KSNP1*KV11)*A(17)*FLAG - KKIL*(ANC/(ANC+ANC50))*(1-A(17)/(A(17)+B50))*A(17)
; S2, Pre-existing resistant bacteria
```

```
DADT(18)= (KSNP1*KV1)*A(15)*FLAG - (KNPS2*KV2)*A(18)-(KK+DRUGS)*(A(18))-
KKIL*(ANC/(ANC+ANC50))*(1-A(18)/(A(18)+B50))*A(18)
               ; NP, non-plateable

DADT(19)=-KK*(A(19)) + SR2*(A(17)) - KKIL*(ANC/(ANC+ANC50))*(1-
A(19)/(A(19)+B50))*A(19)
            ; R2, resting Pre-existing resistant bacteria
DADT(20)= (KSNP1*KV11)*A(17)*FLAG - (KNPS2*KV22)*A(20)-(KK+DRUGS2)*(A(20))-
KKIL*(ANC/(ANC+ANC50))*(1-A(20)/(A(20)+B50))*A(20)

;;----------------------------------RESISTANT STRAIN-----------------------------
-----;;
DADT(21)= KGS*(A(21))-(KK+DRUGS1)*(A(21)) - SR*(A(21)) +(KNPS2*KV21)*A(24) -
(KSNP1*KV110)*A(21)*FLAG - KKIL*(ANC/(ANC+ANC50))*(1-A(21)/(A(21)+B50))*A(21)
      ; S, suspectible
DADT(22)=-KK*(A(22)) + SR*(A(21)) - KKIL*(ANC/(ANC+ANC50))*(1-
A(22)/(A(22)+B50))*A(22)            ; R, resting

DADT(23)= KGS2*(A(23))-(KK+DRUGS21)*(A(23)) - SR2*(A(23)) + (KNPS2*KV221)*A(26)
-(KSNP1*KV111)*A(23)*FLAG- KKIL*(ANC/(ANC+ANC50))*(1-A(23)/(A(23)+B50))*A(23)
  ; S2, Pre-existing resistant bacteria
DADT(24)= (KSNP1*KV110)*A(21)*FLAG - (KNPS2*KV21)*A(24)-(KK+DRUGS1)*(A(24))-
KKIL*(ANC/(ANC+ANC50))*(1-A(24)/(A(24)+B50))*A(24)
               ; NP, non-plateable

DADT(25)=-KK*(A(25)) + SR2*(A(23))- KKIL*(ANC/(ANC+ANC50))*(1-
A(25)/(A(25)+B50))*A(25)
             ; R2, resting Pre-existing resistant bacteria
DADT(26)= (KSNP1*KV111)*A(23)*FLAG - (KNPS2*KV221)*A(26)-(KK+DRUGS21)*(A(26))-
KKIL*(ANC/(ANC+ANC50))*(1-A(26)/(A(26)+B50))*A(26)


$ERROR
AA1 = A(1)                                                ; AMT/Arteries
AA2 = A(2)                                                ; AMT/Veins
AA3 = A(3)                                                ; AMT/Lungs
AA4 = A(4)                                                ; AMT/Brain
AA5 = A(5)                                                ; AMT/Heart
AA6 = A(6)                                                ; AMT/Skin
AA7 = A(7)                                                ; AMT/Muscle
AA8 = A(8)                                                ; AMT/Adipose
AA9 = A(9)                                                ; AMT/Spleen
AA10= A(10)                                               ; AMT/GIO
AA11= A(11)                                               ; AMT/Liver
AA12= A(12)                                               ; AMT/Kidney
AA13= A(13)                                               ; AMT/Rest
;;-------------------------------------------(PK)PD---------------------------
---;;

AA15=A(15)
AA16=A(16)
AA17=A(17)
;AA18=A(18)
AA19=A(19)
;AA20=A(20)

AA21=A(21)
AA22=A(22)
AA23=A(23)
;AA24=A(24)
AA25=A(25)
;AA26=A(26)
```

```
TOT2=AA15+AA16+AA17+AA19
ATOT2=TOT2
TOT3=AA21+AA22+AA23+AA25
ATOT3=TOT3
ATOT4=ATOT2+ATOT3

CC1 = A(1)/VART*FUP                        ; AMT/Arterial volume
CC2 = A(2)/VVEN*FUP                        ; AMT/Venous volume
CC3 = A(3)/VLUN*0.79                       ; AMT/Lung volume
CC4 = A(4)/VBRA*0.79                       ; AMT/Brain volume
CC5 = A(5)/VHRT*0.79                       ; AMT/Heart volume
CC6 = A(6)/VSKN*0.65                       ; AMT/Skin volume
CC7 = A(7)/VMUS*0.79                       ; AMT/Muscle volume
CC8 = A(8)/VADI*0.79                       ; AMT/Adipose volume
CC9 = A(9)/VSPL*0.79                       ; AMT/Spleen volume
CC10= A(10)/VGIO*0.67                      ; AMT/GIO volume
CC11= A(11)/VHEP*0.79                      ; AMT/Hepatic volume
CC12= A(12)/VKID*0.79                      ; AMT/Kidney volume
CC13= A(13)/VRES*0.79                      ; AMT/Rest volume

STRT=1
IF (PER.GT.1) STRT=2

IPRD2 = A(2)/VVEN
IF (IPRD2.LE.0.000001) IPRD2=0.000001
IF (TOT2.LE.0.001) ATOT2=0.001
IF (TOT3.LE.0.001) ATOT3=0.001

IF (CMT.EQ.2) THEN
IPRED = LOG(IPRD2)
IRES = DV-IPRED
W = SQRT(SIGMA(1))
IWRES = IRES/W

Y = IPRED + EPS(1)
ENDIF

;; ---------------------------- Initial estimates Theta PBPK -------------------
---------- ;;

$THETA  2.60                                      ;1 ~CLH
$THETA  0.674                                     ;2~RSEC Renal secretion
$THETA  1.20                                      ;3 ~KLUN
$THETA  -0.257                                    ;4 ~KBRA
$THETA  1.30                                      ;5 ~KHRT
$THETA  -0.335                                    ;6 ~KSKN
$THETA  0.0229                                    ;7 ~KMUS
$THETA  -0.885                                    ;8 ~KADI
$THETA  0.668                                     ;9 ~KSPL
$THETA  1.21                                      ;10 ~KGIO
$THETA  1.27                                      ;11~KHEP
$THETA  2.09                                      ;12~KKID
$THETA  1.35                                      ;13~KRES

;; ---------------------------- Initial estimates Theta PKPD -------------------
---------- ;;
$THETA  (1,1.70)                                  ; 14. KGS growth
$THETA  (0 FIX)                                   ; 15. Parameter not used
$THETA  (0,5.24)                                  ; 16. EMAX
$THETA  (0,0.0368)                                ; 17. EC50 347
$THETA  (0,91.6)                                  ; 18. EC501 707
                                                '
$THETA  (0.5,1.98)                                ; 19. GAM
```

```
$THETA  (0 FIX)                                    ; 20. Parameter not used
$THETA  (0,0.0186)                                 ; 21. FEED_PC
$THETA  (0.18,0.344)                               ; 22. KGS2 growth2
$THETA  (0  FIX)                                   ; 23. Parameter not used
$THETA  (0,1.25)                                   ; 24. EC502
$THETA  (0  FIX)                                   ; 25. Parameter not used
$THETA  (0,0.819)                                  ; 26. MUT start conc pre-
existing resistant347
$THETA  (0  FIX)                                   ; 27. Parameter not used
$THETA  (0,5.83)                                   ; 28. Knp1
$THETA  (0,0.240)                                  ; 29. NP50
$THETA  (0,0.174)                                  ; 30. Knp2
$THETA  (2,5.34)                                   ; 31. MTIME
$THETA  (0,20) FIX                                 ; 32. Hill factor np.

;; ------------------------------ THETA prior values ------------------------
;;
$THETA  1.97 FIX                                   ;1 ~CLH
$THETA  0.57 FIX                                   ;2~RSEC Renal secretion
$THETA  1.2  FIX                                   ;3 ~KLUN
$THETA  -0.26 FIX                                  ;4 ~KBRA
$THETA  1.3 FIX                                    ;5 ~KHRT
$THETA  -0.33 FIX                                  ;6 ~KSKN
$THETA  0.46 FIX                                   ;7 ~KMUS
$THETA  -0.80 FIX                                  ;8 ~KADI
$THETA  0.67 FIX                                   ;9 ~KSPL
$THETA  1.22 FIX                                   ;10 ~KGIO
$THETA  1.3 FIX                                    ;11~KHEP
$THETA  2.1 FIX                                    ;12~KKID
$THETA  1.02 FIX                                   ;13~KRES

;; ------------------------ OMEGA initial estimates ------------------------
;;
$OMEGA  0.316                                      ;1 ~IIV_CLH
$OMEGA  0.306                                      ;2 ~IIV_KP

;; -------------------------- Prior Uncertainty in THETAS --------------------
;;
$OMEGA  0.0625  FIX                                          ;1 ~CLNR prior
$OMEGA  0.0625  FIX                                          ;2 ~RSEC prior
$OMEGA  0.0625  FIX                                          ;3 ~KLUN prior
$OMEGA  0.0625  FIX                                          ;4 ~KBRA prior
$OMEGA  0.0625  FIX                                          ;5 ~KHRT prior
$OMEGA  0.0625  FIX                                          ;6 ~KSKN prior
$OMEGA  0.0625  FIX                                          ;7 ~KMUS prior
$OMEGA  0.0625  FIX                                          ;8 ~KADI prior
$OMEGA  0.0625  FIX                                          ;9 ~KSPL prior
$OMEGA  0.0625  FIX                                          ;10~KGIO prior
$OMEGA  0.0625  FIX                                          ;11~KHEP prior
$OMEGA  0.0625  FIX                                          ;12~KKID prior
$OMEGA  0.0625  FIX                                          ;13~KRES prior

; ----------------------------- SIGMA -------------------------- ;;
$SIGMA  0.112                                                ;1~ Prop RES_ERR

$ESTIMATION METHOD=1 INTER POSTHOC NOABORT PRINT=5 MAXEVALS=0
$TABLE ID TIME CC1 CC2 CC3 CC4 CC5 CC6 CC7 CC8 CC9 CC10 CC11 CC12 CC13 CL DRUGS
DRUGS1 DRUGS2 DRUGS21
      CLH CLR TOTA TOT ATOT2 ATOT3 CUMDOSE NOPRINT ONEHEADER FILE=mytab18
```
